# Serological Diversity of *Dichelobacter nodosus* in German Sheep Flocks

**DOI:** 10.3390/ani12060753

**Published:** 2022-03-17

**Authors:** Monia Budnik, Ann-Kathrin Struck, Julia Storms, Anna Wirth, Jörg Jores, Peter Kuhnert, Ottmar Distl

**Affiliations:** 1 Institute of Animal Breeding and Genetics, University of Veterinary Medicine Hannover (Foundation), 30559 Hannover, Germany; monia.lara.budnik@tiho-hannover.de (M.B.); ann-kathrin.kachler@tiho-hannover.de (A.-K.S.); julia.storms@tiho-hannover.de (J.S.); anna.maria.wirth@tiho-hannover.de (A.W.); 2 Institute of Veterinary Bacteriology, Vetsuisse Faculty, University of Bern, 3012 Bern, Switzerland; joerg.jores@vetsuisse.unibe.ch (J.J.); peter.kuhnert@vetsuisse.unibe.ch (P.K.)

**Keywords:** footrot, sheep, serogroups, *Dichelobacter nodosus*, Germany, serogroup-specific PCR

## Abstract

**Simple Summary:**

Footrot is an infectious hoof disease in sheep, caused by the bacterium *Dichelobacter nodosus*. The antigentic variation of the fimbrial proteins resulted in the description of up to ten serogroups (A–I and M). Vaccines against footrot target these fimbrial variants. Commercial vaccines are covering nine serogroups but have low efficacy compared to vaccines based on two serogroups. Therefore, our study investigated the prevalence and distribution of the nine serogroups A–I in German sheep flocks with the aim to detect the predominant serogroups guiding optimized vaccines based on two serogroups. Serogroup A was most common in our study, followed by serogroups B, H and C. More than one-third of the animals showed more than one serogroup. In flocks, we found, on average, 3.10 serogroups in a range of one to six. The nine serogroups were widely distributed across the flocks, with 50 different combinations across the 83 flocks investigated. The lack of two predominant serogroups in Germany impairs the nationwide protection against footrot by the usage of more efficient vaccines based on two serogroups and requires tailor-made flock-specific vaccines.

**Abstract:**

Footrot is one of the major causes of lameness in sheep and leads to decreased animal welfare and high economic losses. The causative agent is the Gram-negative anaerobic bacterium *Dichelobacter nodosus*. The prevalence of *D. nodosus* in 207 sheep flocks across Germany was 42.9%. Based on the sequence variation in the type IV fimbrial gene *fimA*, *D. nodosus* can be subdivided into ten serogroups (A–I and M). There are commercially available vaccines covering nine serogroups, but the efficacy is low compared to bivalent vaccines. The aim of this study was to investigate the diversity of serogroups in Germany at the flock and animal levels. In total, we detected at least one serogroup in 819 samples out of 969 *D. nodosus*-positive samples from 83 flocks using serogroup-specific singleplex PCR for the serogroups A–I. Serogroup A was most prevalent at the animal level, followed by serogroups B, H and C. At the flock level, serogroups A and B had the highest prevalence, each with 64%, but only 40% of flocks had both. The average number of serogroups per animal was 1.42 (range one to five) and, per flock, 3.10 (range one to six). The serogrouping showed within-flock specific clusters but were widely distributed, with 50 different combinations across the flocks. The factors associated with the number of serogroups per animal and single serogroups were the load of *D. nodosus*, footrot score, sheep breed and flock. Our results indicate that efficient vaccination programs would benefit from tailor-made flock-specific vaccines and regular monitoring of circulating serotypes in the flock to be able to adjust vaccine formulations for nationwide progressive control of footrot in Germany.

## 1. Introduction

Ovine footrot is a contagious disease, which is primarily caused by the Gram-negative anaerobic bacterium *Dichelobacter nodosus* [1,2]. It is one of the major causes of lameness in sheep and leads to weight loss and reduced wool production [3]. Furthermore, reduced lambing percentages, reduced lambing survival and higher veterinary costs were reported for ovine footrot [4]. The clinical signs vary from mild interdigital dermatitis to underrunning of the hoof horn in severe cases [5]. Footrot is very painful and causes lameness, which implicates that the disease needs to be recognized as an issue for animal welfare [6].

The severity of the clinical signs result from the virulence of the *D. nodosus* strain, moist environmental conditions and immune response of the host [7].

Currently, ten serogroups named A–I and M are described for *D. nodosus*. These serogroups are based on their type IV fimbrial subunit antigen encoded by *fimA* [8]. The fimbrial antigen was shown to be a major protective antigen; however, there is no cross-protection between different serogroups [9].

The commercially available vaccine *Footvax* (MSD Animal Health, Kenilworth, NJ, USA) is a multivalent vaccine containing serogroup antigens A–I [10]. For this vaccine, a variable response and only partial protection were reported [9,11]. Mono- or bivalent vaccines generated better results in controlling the clinical disease [9,12]. With an increasing number of *D. nodosus* fimbrial antigens in the vaccine formulation, the protective effect decreases linearly [13]. Schwartzkoff et al. explained this effect with antigenic competition between different serogroup antigens [14]. Therefore, monovalent products initiate a better immune response and higher antibody titres than multivalent vaccines. Due to lower and less persistent antibody titers, the duration of the protective effect was lower in multicomponent products than in bivalent vaccines; under a severe challenge, it only offered protection for less than ten weeks [14,15]. Therefore, multivalent vaccines need to be boostered more often than bivalent vaccines [15]. Furthermore, autogenous vaccines contain milder adjuvants; therefore, they cause a lower number of local reactions than *Footvax*, which is an animal welfare issue [16].

The effect of antigenic competition was also reported when vaccinating with two bivalent vaccines concurrently [17]. An inter-vaccination interval of two months for the usage of bivalent vaccines containing different antigens was suggested to avoid antigenic competition [15].

To the best of our knowledge, there was only one study investigating *D. nodosus* serogroups in Germany [18]. This study comprised 82 *D. nodosus* isolates from nine flocks located in Southwest Germany and revealed serogroup B to be predominant in all flocks [18].

The current study is a follow-up to a previous study, where the prevalence of *D. nodosus* was determined in 207 sheep flocks across Germany using 9243 swab samples from the interdigital skin of feet. The prevalence of *D. nodosus* reported was 42.9% [19]. The aim of this study was therefore to investigate the distribution and prevalence of the nine serogroups A–I across Germany based on serogroup-specific singleplex PCRs. The samples were obtained during a previous study on the prevalence of *D. nodosus* [19] and included flocks with at least four *D. nodosus*-positive samples. We tested the distribution of the serogroups at the flock and animal levels by regions, footrot scores, virulence of *D. nodosus* strains, load of *D. nodosus* and further flock-specific factors. Here, we present the most representative study of serological diversity of *D. nodosus* for a large number of flocks across Germany. The data collected in this study could be useful in the support ofvaccination programs and to determine the best vaccination strategy.

## 2. Materials and Methods

### 2.1. Ethical Approval

This study was carried out according to the approval from the Institutional Animal Care and Use Committee (IACUC) (33.19-42502-05-19A414) and the respective state veterinary offices from the different German states. The European Union guidelines for animal care and the Guidelines of Good Veterinary Practices were followed.

### 2.2. Sample Collection

The project was announced broadly in meetings of sheep breeding organizations all over Germany, discussion rounds of sheep breeders and veterinary practitioners, sheep breeder’s journals (Schäferbrief and Schafzucht) and was published on our website (https://www.tiho-hannover.de/kliniken-institute/institute/institut-fuer-tierzucht-und-vererbungsforschung/forschung/forschungsprojekte-schaf/moderhinke-mores, accessed on 11 March 2022).

All sheep owners were invited to participate regardless of the clinical footrot status of their flocks. Before participation, we obtained informed written consent from all farmers. All farmers received a questionnaire to give us some background information. The questionnaire included information about their herdbook membership, flock size, sheep breeds, other livestock species present on the farm, reports of previous outbreaks of footrot and strategies for the treatment of diseased animals.

**Figure 1:**
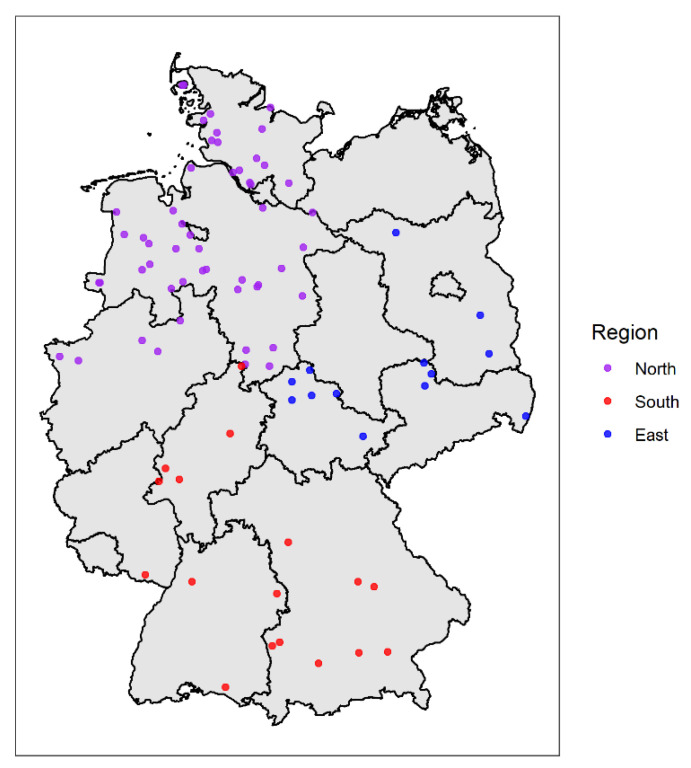
Geographical location of the 83 sheep flocks in Germany from which samples were used to determine the serogroups. Purple: Northern Germany, blue: Eastern Germany and red: Southern Germany.

For the investigation of the geographic distribution of the serogroups, the participating farms were evaluated depending on their location to the three study areas. The study areas were Northern Germany (federal states Schleswig-Holstein, Lower Saxony and North Rhine-Westphalia); Eastern Germany (federal states Brandenburg, Saxony, Saxony-Anhalt and Thuringia) and Southern Germany (Federal states Bavaria, Baden-Württemberg, Hesse and Rhineland-Palatinate) (Figure 1). Lesions were classified by the footrot scoring system of the Swiss Consulting and Health Service for Small Ruminants (Supplementary Table S1) [20].

Data sampling was from January 2019 to September 2020. Dry and clean cotton swabs (Sarstedt, Nümbrecht, Germany) in a tube with a screw top and without a transport medium were used for sampling. The interdigital space between the toes of each sheep was swabbed along its entire length, then the swab was rotated 90° and used for the second interdigital space. This was repeated until all four feet were sampled. We collected samples of 9243 sheep from 207 flocks, with flock sizes ranging from 10 to 2400 sheep.

On arrival, swabs were transferred to −20 °C storage until processing.

### 2.3. Detection of D. nodosus by qPCR

For the DNA extraction from the cotton swabs, the Qiagen DNeasy Blood and Tissue Kit (Qiagen, Hilden, Germany) was used according to the manufacturer’s protocol.

The DNA samples were tested using qPCR to discriminate benign and virulent *D. nodosus* strains based on the *aprV2/aprB2* gene polymorphisms following the protocol of Stäuble et al. [19,21]. The 25-µL reaction mixture containing 22.5-µL TaqMan Fast Advance MasterMix (Life Technologies, Thermo Fischer Scientific, Waltham, MA, USA) and 2.5 µL of the extracted sample DNA were pipetted in duplicates into a 96-well plate. Two non-template samples (pyrogen-free water) were used as negative controls for each qPCR run. Positive controls were the type strain ATCC 25549T and the field isolate JF5922 provided by the Institute of Veterinary Bacteriology, University of Bern, Bern, Switzerland [22]. The amplification was carried out in a 7500 Real-Time PCR System (ABI, Thermo Fischer Scientific, Waltham, MA, USA).

For the interpretation of the results, we used QuantStudio 3 System Software (Thermo Fisher Scientific, Waltham, MA, USA) with the threshold set at 0.065. The threshold of the Ct-value for a sample being positive was set at <40.

### 2.4. Selection of Samples for Serotyping

The samples taken for the serogroup-specific PCR in this follow-up study were selected out of the samples that were tested positive for a *D. nodosus* load in qPCR. A minimum of four *D. nodosus*-positive samples per flock was required for flocks to be included in the present study. Of the 207 flocks sampled, in 59 flocks, *D. nodosus* was not detectable, and less than four *D. nodosus*-positive samples were available in a further 65 flocks. Consequently, samples from 83 flocks with a minimum of four samples were eligible for determination of the serogroups. In flocks with a larger number of *D. nodosus*-positive samples, we used more samples but not more than 25 samples per flock, resulting in 969 samples with a median number of 10 samples per flock.

### 2.5. Serotyping by Serogroup-Specific Singleplex PCR

The 969 samples were tested in 96-well plates separately for each of the nine serogroups A–I using primers published by Dhungyel et al., one common forward primer and nine specific reverse primers [23]. Each reaction included 25 µL and was composed of MyTaq Red Mix (Bioline, London, UK) (12.5 μL), forward primer (0.5 μM) and reverse primer (0.5 μM). The amplification cycles consisted of an initial denaturation at 94 °C for 4 min, followed by 94 °C for 30 s, 60 °C for 30 s and 72 °C for 30 s for 7 cycles; 94 °C for 30 s, 58 °C for 30 s and 72 °C for 30 s for 35 cycles and a final extension at 72 °C for 4 min. PCRs were performed on a SensoQuest Labcycler (SensoQuest, Göttingen, Germany). Each PCR reaction included a negative (no template) and a positive control, the latter based on validated PCR products from each serogroup and were normalized to 20 ng/µL. PCR products were analyzed on 3% agarose gels stained with ethidium bromide and visualized under ultraviolet light.

### 2.6. Statistical Analysis

Statistical analysis of the data was performed using SAS, version 9.4 (Statistical Analysis System, Cary, NC, USA). Chi-square tests and exact binomial 95% confidence intervals were calculated with the SAS procedure FREQ. We used the SAS procedure GLIMMIX to evaluate associations for the number of serogroups per animal and prevalence of each serogroup with a random flock within a region effect and, further, fixed effects. These fixed effects were *D. nodosus* strains per flock (*aprV2*, *aprB2* or *aprV2/aprB2*); load of *D. nodosus* (determined by log Ct-values); footrot score; region; herdbook member; breed; flock size; goats, cattle, horses or donkeys on the farm; footrot within the last 12 months; treatment of diseased sheep with antibiotics, footbaths and vaccines for footrot within the last 12 months; footrot within the last 3–10 years; treatment of diseased sheep with antibiotics, footbaths and vaccines for footrot within the last 3–10 years and the number of animals tested for serogroups (Supplementary Table S2). The models employed to test for single fixed effects or covariates always contained the random effect of flock within region, because this random effect was significant for all dependent variates in all models applied. The final generalized mixed linear model for the number of serogroups per animal with a multinomial distribution function and a cumulative logit as the link function included flock within region as a random effect, load of *D. nodosus* as a covariate and the fixed effects of region and treatment of diseased sheep with antibiotics within the last 12 months. The other effects were not significant using stepwise forward and backward selections. The final models for the prevalence of each serogroup are given in Supplementary Table S3.

A mixed linear multivariable model was employed to estimate the effects on the load for all *D. nodosus* and only for *aprV2*-positive strains. The log mean Ct-values from the competitive qPCR specific for *aprV2* and *aprB2* of *D. nodosus* were applied. The *D. nodosus* load was calculated as log(40-Ct+1), resulting in higher values for lower Ct-values, indicative of a higher *D. nodosus* load. The mixed linear model included flock within region as a random effect and fixed effects for footrot scores of the levels 0, 1, 2 and 3–5; the serogroups A–I as each one linear regression; a region with three factors (north, east and south); herdbook membership (yes or no) and sheep breeds, including Dorper, Leine, German Merino Mix, German Merino, German Blackheaded Mutton, Suffolk, Swifter, Texel, German Grey Heath, German White Heath and other breeds. Breeds with less than 25 animals or present in less than 2 flocks were subsumed under other breeds.

## 3. Results

All serogroups investigated in our study (A–I) were detected. We obtained a total of 1163 positive PCR results across the nine singleplex PCR assays. Positive PCR results were obtained for 819 animals from 83 sheep flocks. In 150 animals that were tested positive for *aprV2* and/or *aprB2* strains of *D. nodosus* before, no visible PCR band indicative of a specific serotype was detected. In all the examined flocks, serogroups were detected. A summary about flock-level results for the serogroups, *D. nodosus* strains and footrot scores is given for each flock in Supplementary Table S4.

### 3.1. Prevalence of Serogroups on the Flock and Animal Levels

**Figure 2:**
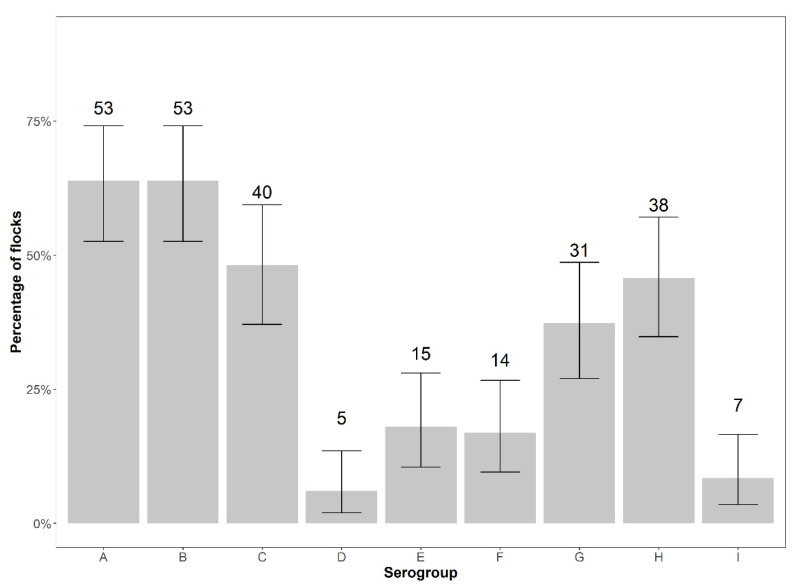
Number and percentage of flocks positive for serogroups A–I of *D. nodosus* with exact binomial 95% confidence intervals.

The most prevalent serogroups on the flock level were serogroups A and B, as each detected in 53 flocks (63.86%). The least prevalent were serogroups D and I, detected in five (6.02%) and seven flocks (8.43%), respectively (Figure 2).

**Figure 3:**
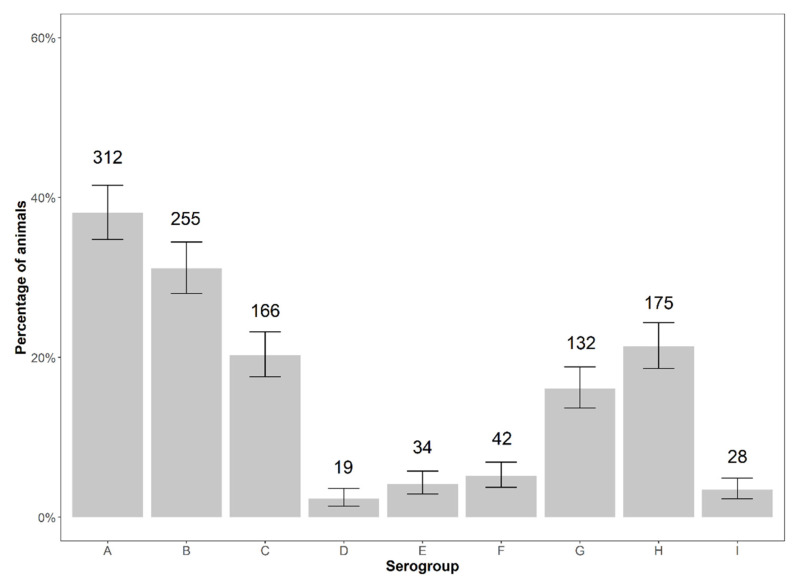
Number and percentage (referring to 819) of animals positive for serogroups A–I of *D. nodosus* with exact binomial 95% confidence intervals.

The most prevalent serogroup on the animal level was serogroup A with 312 positive animals (38.10%), followed by serogroup B with 255 animals (31.14%) and H with 175 animals (21.37%) (Figure 3).

### 3.2. Number of Serogroups Detected at the Flock and Animal Levels

**Figure 4:**
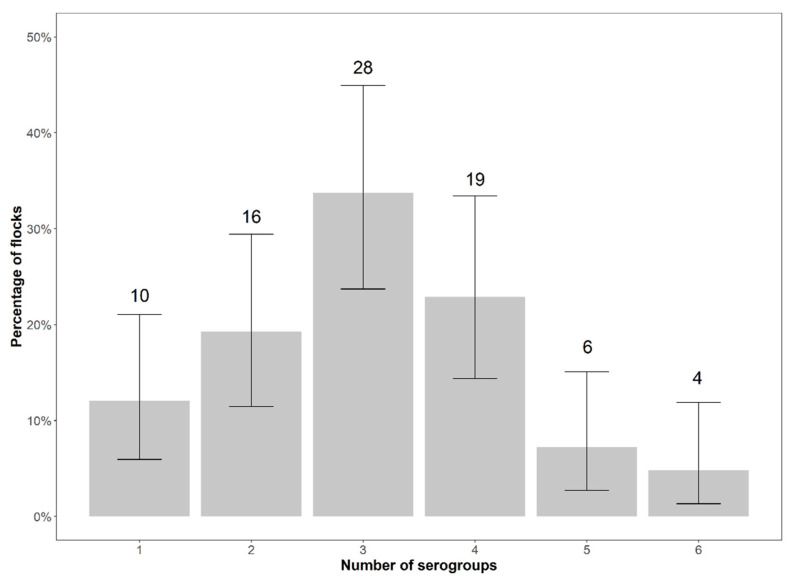
Number and percentage of flocks by number of serogroups detected in the flocks with exact binomial 95% confidence intervals.

The number of serogroups per flock ranged from one to six. In 28 flocks (33.74%), three serogroups were detected; 19 flocks (22.89%) had four serogroups, 16 flocks (19.28%) had two serogroups, ten flocks (12.05%) had a single serogroup and four flocks (4.82%) had six serogroups present at the same time. On average, 3.10 serogroups per flock were detected, and a frequency of 87.95% of the flocks had more than one serogroup present (Figure 4).

**Figure 5:**
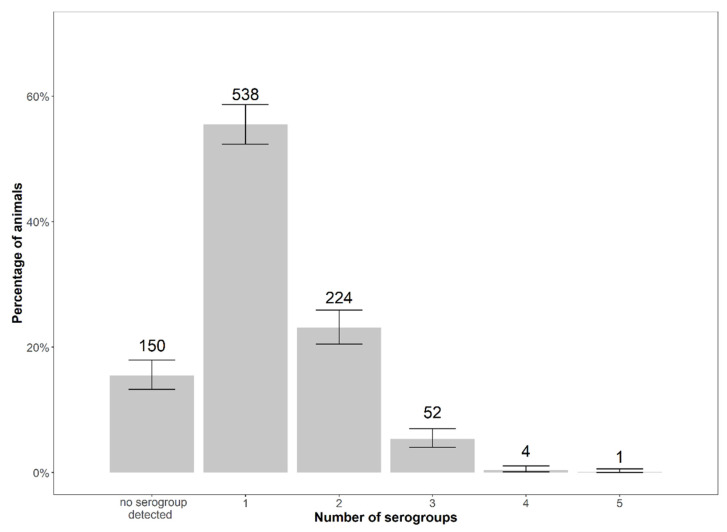
Number and percentage (referring to 969) of animals by number of serogroups detected per animal with exact binomial 95% confidence intervals.

The number of serogroups detected per animal ranged from one to five. We were not able to determine a serogroup in 150 animals (15.45%). The majority of the 819 typeable diagnostic samples (538 samples) contained one serogroup (65.69%). In 27.35% (224 samples), two serogroups were detected. On one animal (0.12%), five serogroups were detected. The average number of serogroups was 1.42 serogroups per animal, as 1163 positive singleplex serogroup PCR results were obtained in 819 samples (Figure 5).

### 3.3. Distribution of Serogroups and Number of Serogroups per Animal by aprV2- and aprB2-Positive Strains of D. nodosus

*AprB2*-positive strains of *D. nodosus* were determined in 55 samples (6.72%) with a serogroup detected, and both strains, *aprV2* and *aprB2*, were determined in 28 samples (3.42%). In *aprV2*-positive strains, all serogroups A–I were detected. In samples containing only the *aprB2*-positive strains, serogroups D and I were not detected, while in samples containing both strains, serogroups D and E were not detected. Serogroups A and B were predominant in samples with *aprV2*-positive strains, as well as in samples with *aprB2*-positive strains (Table 1). Significant differences for serogroup prevalence among the *aprV2*- and *aprB2*-positive strains were observed for serogroups G (*p* = 0.04) and H (*p* = 0.004).

The number of serogroups per animal ranged from one to three in samples containing *aprB2*-positive strains and from one to four in samples containing both strains. One serogroup per sample was most frequent, with 71.88% of the samples containing *aprB2*-positive strains and 58.07% of the samples containing both strains (Table 2). The number of serogroups per animal was not equally distributed among *D. nodosus* strains (*p* = 0.011; χ^2^ = 19.74 with eight df (degrees of freedom)). Testing the animals with each one or two serogroups versus all the others gave significant differences among the *D. nodosus* strains (*p* = 0.015; χ^2^ = 8.43 with two df and *p* = 0.002; χ^2^ = 12.76 with two df).

### 3.4. Distribution of Serogroups and Number of Serogroups in Three Regions of Germany

The different serogroups of *D. nodosus* were widely dispersed across the different regions sampled. All nine tested serogroups were detected in the three different regions. At the animal level, serogroup A was the most prevalent in Northern (42.92%) and Eastern Germany (41.40%), while, in Southern Germany, it was serogroup B (43.86%).

The four most prevalent serogroups in our sample, A, B, C and H, together amounted to 81.7% of the total number of serogroups from Northern Germany (728), 84.1% of the total number of serogroups from Eastern Germany (195) and 62.1% of the total number of serogroups from Southern Germany (240). The prevalence of serogroups A, C, E, F and G were significantly different among the regions at *p*-values <0.001 (χ^2^ = 28.95, two df), <0.001 (χ^2^ = 27.21, two df), 0.005 (χ^2^ = 10.79, two df), 0.026 (χ^2^ = 7.26, two df) and < 0.001 (χ^2^ = 34.77, two df), respectively.

In the samples from Southern Germany, there is a higher prevalence of serogroup G (32%) compared to Northern (11.91%) and Eastern Germany (11.47%) (Table 3).

When the three regions were compared at the flock level, the results were similar to those at the animal level (Supplementary Table S5).

The number of serogroups per animal ranged from one to three in animals from Eastern and Southern Germany, while four and five serogroups per sample were found in the animals from Northern Germany. Three serogroups per animal showed the highest prevalence, with 6.92% in Northern Germany as well (Table 4). The number of serogroups was significantly unequally distributed among the regions (*p* < 0.001; χ^2^ = 27.23, eight df), as well as when comparing animals with one (*p* < 0.001; χ^2^ = 17.80, two df), two (*p* = 0.002; χ^2^ = 12.37, two df) or more than two (*p* = 0.003; χ^2^ = 11.82, two df) serogroups against all other animals with a serogroup determined.

### 3.5. Distribution of Serogroups and Number of Serogroups per Animal by Clinical Footrot Score

For 887 animals, footrot scores on a scale from zero to five were collected (Supplementary Table S1). For 741 animals, footrot scores and serotype data were available; consequently, in 146 animals, there were clinical data but no information on serotypes.

Most of the selected samples for the investigation of serogroups scored one (36.98%), followed by scoring zero (29.31%) and scoring two (18.26%).

In these 741 samples, we detected, in total, 1054 serogroups. Serogroups A–I, except for serogroup D, were determined in animals with all six footrot scores. Serogroup D was only detected in animals with scores one to three (Table 5). The prevalence of serogroups A, C, D, E, F, G, H and I was significantly different among the footrot scores at *p*-values <0.001 (χ^2^ = 27.77, five df), 0.004 (χ^2^ = 17.28, five df), 0.037 (χ^2^ = 11.88, five df), <0.001 (χ^2^ = 21.82, five df), 0.016 (χ^2^ = 13.97, five df), <0.001 (χ^2^ = 24.39, five df), 0.047 (χ^2^ = 11.24, five df) and 0.004 (χ^2^ = 17.45, five df), respectively.

The number of serogroups per animal ranged from one to three within all the scores. Four and five serogroups were detected in samples from sheep with scores of one to three. For all scores, one serogroup per animal was most common, with 49.23–65.63% of the total number of animals with the respective scores (Table 6). There were no significant differences in the distribution of the number of serogroups among the footrot scores.

### 3.6. Generalized Linear Multivariable Model for Number of Serogroups and Serogroups

The final model showed an increase in number of serogroups, with a higher load of *D. nodosus* (0.0953 ± 0.0281) and a higher number of serogroups with the use of antibiotics within the last 12 months (0.1875 ± 0.0605) compared to no use of antibiotics. Significant effects for the prevalence of serogroups are shown in Supplementary Table S3. A load of *D. nodosus* had a significant effect on serogroups A, C, F and G. Breed was significant for serogroups A, B and C. Footrot score was significantly related to the prevalence of serogroups A, B, E and H. The random flock within region effect was significant for all dependent variates and explained between 21.9 (number of serogroups) and 66.1% (serogroup H) of the variance.

### 3.7. Load of D. nodosus

We used a mixed linear multivariable model to estimate the effects on the load for all *D. nodosus* strains and only for *aprV2*-positive strains. With increasing footrot scores, the loads of all and *aprV2*-positive strains of *D. nodosus* significantly increased (Table 7). A significant lower load of all and *aprV2*-positive strains of *D. nodosus* was associated with serogroups A and C. Animals with serogroup G showed a significantly higher increase in the loads of all *D. nodosus*. The variance among flocks within region was significantly different from zero and explained 56 and 60% of the total variation for the loads of the all and *aprV2*-positive strains of *D. nodosus*.

### 3.8. Prevalence of Serogroup Combinations across Flocks

There were 50 combinations of serogroups across the flocks (Supplementary Table S6). In 33 flocks (39.76%), eight different combinations were observed more than twice. In seven of the eight combinations, serogroup A was present, which reflects that this is the serogroup with the highest prevalence. The combination of serogroups A, B and H together in one flock occurred most frequently, namely in seven flocks.

In 10 flocks (12.05%), only one serogroup was found. Serogroups A, B, C and H were found as singletons twice and serogroups F and G each once. Serogroups D, E and I were only detected together with other serogroups. Only four combinations of serogroups were each present in more than three flocks, with one combination (A, B, H) in seven flocks.

### 3.9. Theoretical Protection of Bivalent Vaccines in Germany

According to the distribution of the serogroup combinations in our study, a bivalent vaccine containing serogroups A and B would fully protect six of the examined flocks (7.23%) and partially protect 67 of the examined flocks (80.72%). Consequently, 87.95% of the flocks could gain full protection by the usage of such a bivalent vaccine. Similar results could be expected when using a bivalent vaccine containing serogroups B and H: eight flocks (9.64%) would be fully protected, and 56 flocks (67.47%) would be partially protected.

When using two bivalent vaccines containing the four most prevalent serogroups: A, B, C and H one after another with an adequate inter-vaccination interval, 37 of the examined flocks (44.58%) could gain full protection. In addition, 43 flocks (51.81%) would be partially protected by the usage of this vaccine. In only three flocks (3.61%), none of these four serogroups were detected and hence, no protection would be provided.

## 4. Discussion

Our study confirmed the presence of at least nine different *D. nodosus* serogroups in Germany. In that respect, sheep flocks were characterized by different serogroup combinations, as well as a wide combinatorial diversity of serogroups across Germany. In 57/83 flocks, different serogroup combinations were found. The prevalence of the serogroups varied between flocks and geographical locations with 50 different serogroup combinations across the flocks. The large proportion of variance between flocks was indicative of serogroup patterns specific for flocks. Therefore, a single uniform bivalent vaccine does not present the best vaccine solution, and tailor-made vaccines to be amended for individual flocks are indicated to control footrot via the vaccine route. The use of a commercial vaccine has to be flanked via other control measures, such as regular footbaths to control footrot.

The sheep farmers in the study participated voluntarily. Therefore, the sample collection was not completely randomized. In addition, due to management and logistics of the sample collections, there was no equal distribution of the flocks across the German states.

For the detection of *D. nodosus*, we used the qPCR method published by Stäuble et al. [21]. This sensitive and specific method for the detection of *D. nodosus* has been applied successfully in other studies [24,25,26,27]. The method we used for the serogroup-specific PCR was published by Dhungyel et al. [23], which supplied sensitive results in recent studies [28,29,30].

A total of 150 samples tested negative, showing no serogroup-specific PCR amplification. It is possible that part of the negative samples contained serogroup M, which currently cannot be detected by PCR, as suitable primers could not be identified. Therefore, serogroup M is generally not tested in such studies [27,28,30]. For the identification of serogroup M, culturing and slide agglutination would be necessary. In Australia, the prevalence of serogroup M was thereby found to be 40% [31]. In Nepal, Ghimire et al. found serogroup M at a prevalence of 6.2% [32]. In Norway, serogroup M was not detected in sheep samples but in cattle [33].

In our study with samples of sheep flocks across Germany, serogroup A was most prevalent, followed by serogroups B, H and C. At the flock level, the same four serogroups were most frequently observed.

A German study on 66 typeable strains out of 82 isolated strains from nine footrot-affected flocks revealed the highest prevalence for serogroup B with 64.4%, followed by C (9.6%) and G (2.4%). For the latter study, *D. nodosus* was cultivated from hoofhorn samples. Other serogroups detected were A, E and H but each only once. Serogroups D, F and I were not identified [18]. Due to the low number of flocks and application of other methods, the results are not comparable to our study results.

Our results are in agreement with studies from Sweden and Norway, where serogroup A was predominant [29,33]. In Australia, New Zealand, India and Bhutan, serogroup B was most frequently observed [28,34,35,36], while, in Great Britain, serogroups B and H [27,30,37,38] were the most prevalent.

On average, each sample contained 1.42 serogroups (range one to five) and each flock 3.10 serogroups (median = 3, range one to six). The number of samples tested per flock was not significantly correlated with the outcome of the present study. The number of serogroups detected per flock was slightly higher than in a recent study from the UK [30] and slightly lower than in a report on 24 lowland English sheep farms with clinical footrot with a median of five and a mean value of 4.4 [27]. Comparing the results between our and the latter two studies indicated that, with an increasing number of *D. nodosus*-positive samples per flock, the number of serogroups detected per flock also increased. We found a median of three serogroups per flock with a median of 10 *D. nodosus*-positive samples per flock. Monaghan et al. found a median of five serogroups per flock, with a median of 16 *D. nodosus*-positive samples per flock, and Prosser at al. found a median of two serogroups per flock, with a median of four *D. nodosus*-positive samples per flock [27,30].

The number of serogroups per flock ranged likewise from one to six in studies from Australia and the UK [30,39]. Other studies from the UK reported lower numbers of serogroups per flock [37,38]. This difference may be caused by different methods, sampling designs, sheep breeds investigated and livestock production systems. In the latter mentioned studies, serotyping was performed on cultured isolates, while, in our study and previous reports from the UK [25,28], serotyping was performed directly on swab samples.

The distribution of serogroups on samples containing *aprV2*-positive strains, *aprB2*-positive strains and both strains needs to be reviewed critically because of the low number of samples containing *aprB2*-positive and both strains. In a Swedish study on 78 *D. nodosus* samples, with 66 isolates containing *aprB2*-positive strains and 12 isolates containing *aprV2*-positive strains, serogroups A, B, C, E, G and H were detected in the *aprB2*-positive strain samples, and all samples with serogroup A harbored *aprB2*-positive strains [29]. A Norwegian study on 214 *aprB2*-positive isolates and 305 *aprV2*-positive isolates revealed a greater serogroup diversity of *aprB2*-positive versus *aprV2*-positive isolates [33]. These results are not in agreement with our results, because we found a greater serogroup diversity with *aprV2*-positive than with *aprB2*-positive strains. In *aprB2*-positive strains, serogroups D and I were not detected, but serogroup I was detected on samples with both strains. However, serogroup I has been detected with *aprB2*-positive strains from Norway before [33]. Therefore, the absence of this serogroup is probably due to the low number of samples. Serogroup D has also not been detected with *aprB2*-positive strains in other studies [29,33]. This may indicate that this serogroup occurs only with *aprV2*-positive strains. Probably, this is caused by the low number of samples and that serogroup D is very rare. Our results are not in agreement with a recent study from the UK, which reported serogroups C and E being more likely present in samples with both *aprV2*- and *aprB2*-positive *D. nodosus* strains [27]. In agreement with our study, serogroup H was more likely with *aprV2*-positive *D. nodosus* strains.

The only previous study on *D. nodosus* serogroups in Germany was restricted to nine flocks in Southwest Germany [18]. The present study includes a large number of flocks across Germany and should give a much more detailed insight into the diversity of serogroups than this previous report. All nine serogroups were detected in all three regions of Germany. An overdominance of serogroup B was ruled out in the current study. The participating farmers in our study often purchased sheep from distant locations and did not quarantine sheep after arrival; therefore, the pathogen is widely spread across sheep flocks in Germany [19].

In an Australian study, multivalent and bivalent vaccination strategies were compared, with the result that the duration of protection achieved by multivalent vaccines is less than with bivalent vaccines. Therefore, bivalent vaccines are a good option for the prevention of footrot [15]. We found 50 different combinations of serogroups, and only eight of them occurred more than twice. Similar results were reported by Prosser et al., who found 50 combinations in 138 flocks from the UK [30]. This high amount of different combinations of the serogroups prevents successful vaccination with a single bivalent vaccine. With a bivalent vaccine containing serogroups A and C, six flocks (7.23%) would gain full protection, and 67 flocks (80.72%) would gain partial protection. For the effective usage of one bivalent vaccine in Germany, there is a lack of dominance of two serogroups. Therefore, an effective prophylactic protection needs flock-specific vaccines. An alternative may be the usage of two bivalent vaccines with an adequate inter-vaccination interval, but this approach would need further investigation. In an Australian study, a two-month interval was reported to be sufficient to avoid antigenic competition [15]. In our study with two bivalent vaccines containing the four most prevalent serogroups: A, B, C and H, 80 of the 83 flocks would get full or partial protection.

Our results are restricted to serogroups A–I, as the prevalence of serogroup M could not be estimated. In 37/83 flocks, we found samples with untypeable serogroups. In nine flocks, more than five samples were untypeable, and in 11/83 and 5/83 flocks, more than 30% and 50% of the samples were untypeable, respectively. The pattern of the untypeable serogroups suggests that they are randomly distributed across the flocks and cluster in a few flocks. In these flocks, a further increase in the number of serogroups should be expected.

## 5. Conclusions

In conclusion, *D. nodosus* serogroups are randomly distributed across German sheep flocks. There is a lack of predominance of one or two serogroups in flocks together, which would enable the usage of one bivalent vaccine. Flock-specific vaccination or the use of two bivalent vaccines containing all four most prevalent serogroups in a combination is necessary for a nationwide protection.

## Figures and Tables

**Table 1:** Distribution of the serogroups in samples containing *aprV2*-positive strains, *aprB2*-positive strains and both strains of *D. nodosus*. Number (No) and percentage of animals with the respective serogroup. The percentages refer to the number of animals with the serogroups and the respective strain (*n* = 819).

*D. nodosus*	No of Animals	No (%) of Animals with the Respective Serogroup								
		A	B	C	D	E	F	G	H	I
*aprV2*-positive	736	279 (37.91)	224 (30.44)	153 (20.79)	19 (2.58)	31 (4.21)	38 (5.16)	112 (15.22)	169 (22.96)	26 (3.53)
*aprB2*-positive	55	23 (41.82)	18 (32.73)	7 (12.73)	0 (0)	3 (5.46)	2 (3.64)	11 (20.00)	5 (9.09)	0 (0)
Both	28	10 (35.71)	13 (46.43)	6 (21.43)	0 (0)	0 (0)	2 (7.14)	9 (32.14)	1 (3.57)	2 (7.14)

**Table 2:** Number (No) and percentage of serogroups per animal by *aprV2*-positive strains, *aprB2*-positive strains or both strains of *D. nodosus*. The percentages refer to the total number of animals (*n* = 969) positive for the respective strain.

*D. nodosus*	No of Animals	No (%) of Serogroups per Animal					
		0	1	2	3	4	5
*aprV2*-positive	874	138 (15.79)	474 (54.23)	214 (24.49)	44 (5.03)	3 (0.34)	1 (0.11)
*aprB2*-positive	64	9 (14.06)	46 (71.88)	4 (6.25)	5 (7.81)	0 (0)	0 (0)
Both	31	3 (9.68)	18 (58.07)	6 (19.36)	3 (9.68)	1 (3.23)	0 (0)

**Table 3:** Distribution of the serogroups by the three different regions of Germany at the animal level. Number and percentage of animals with the respective serogroups. The percentages refer to the number of animals with serogroups detected in the respective regions (*n* = 819).

Region	No of Flocks	No of Animals	No (%) of Animals with the Respective Serogroup								
			A	B	C	D	E	F	G	H	I
North	52	487	209 (42.92)	149 (30.60)	126 (25.87)	9 (1.85)	17 (4.49)	33 (6.78)	58 (11.91)	111 (22.79)	16 (3.29)
East	14	157	65 (41.40)	45 (28.66)	19 (12.10)	3 (1.91)	3 (1.91)	4 (2.55)	18 (11.47)	35 (22.29)	3 (1.91)
South	17	175	39 (22.29)	61 (43.86)	20 (11.43)	7 (4.00)	14 (8.00)	5 (2.86)	56 (32.00)	29 (16.57)	9 (5.14)

**Table 4:** Number (No) and percentage of serogroups per animal in the three regions. The percentages refer to the total number of animals tested positive for *D. nodosus* in the respective regions (*n* = 969).

Region	No of Animals	No (%) of Serogroups per Animal					
		0	1	2	3	4	5
North	578	97 (16.78)	291 (50.35)	145 (25.09)	40 (6.92)	4 (0.69)	1 (0.17)
East	193	20 (10.36)	135 (69.95)	29 (15.03)	9 (4.66)	0 (0)	0 (0)
South	198	33 (16.67)	112 (56.57)	50 (25.25)	3 (1.51)	0 (0)	0 (0)

**Table 5:** Distribution of serogroups by animals and footrot scores. Number (No) and percentage of animals with the respective serogroups. The percentages refer to the number of animals with serogroups detected and footrot scores.

Footrot Score	No of Animals	No (%) of Animals with the Respective Serogroups								
		A	B	C	D	E	F	G	H	I
0	187	78 (41.71)	59 (31.55)	45 (24.06)	0 (0)	1 (0.54)	9 (4.81)	25 (13.37)	35 (18.72)	4 (2.14)
1	268	111 (41.42)	101 (37.69)	42 (15.67)	1 (0.37)	13 (4.85)	11 (4.10)	30 (11.19)	65 (24.25)	6 (2.24)
2	152	41 (26.97)	53 (34.87)	49 (32.24)	2 (1.32)	5 (3.29)	4 (2.63)	25 (16.45)	39 (25.66)	4 (2.63)
3	79	15 (18.99)	19 (24.05)	17 (21.52)	3 (3.80)	10 (12.66)	6 (7.60)	13 (16.46)	27 (34.18)	7 (8.86)
4	31	5 (16.13)	6 (19.35)	7 (22.58)	0 (0)	3 (9.69)	4 (12.90)	8 (25.81)	6 (19.36)	4 (12.90)
5	24	6 (25.00)	8 (33.33)	3 (12.50)	0 (0)	1 (4.17)	4 (14.82)	11 (45.83)	2 (8.33)	1 (4.17)

**Table 6:** Number (No) and percentage of serogroups per animal by the clinical footrot scores. The percentages refer to the total number of animals with the respective scores.

Footrot Score	No of Animals	No (%) of Serogroups per Animal					
		0	1	2	3	4	5
0	260	73 (28.08)	128 (49.23)	49 (18.85)	10 (3.85)	0 (0)	0 (0)
1	328	60 (18.29)	174 (53.05)	77 (23.48)	16 (4.89)	1 (0.31)	0 (0)
2	162	10 (6.17)	104 (64.20)	30 (18.52)	15 (9.26)	2 (1.24)	1 (0.62)
3	81	2 (2.47)	46 (56.79)	29 (35.80)	3 (3.70)	1 (1.23)	0 (0)
4	32	1 (3.13)	21 (65.63)	8 (25.00)	2 (6.25)	0 (0)	0 (0)
5	24	0 (0)	15 (62.50)	6 (25.00)	3 (12.50)	0 (0)	0 (0)

**Table 7:** Source of variation, effect estimates with their standard errors (SE) and *p*-values for loads of all and *aprV2*-positive strains of *D. nodosus* from the mixed linear multivariable model.

Source of Variation	All *D. nodosus*		*AprV2*-Positive Strains of *D. nodosus* Only	
	Estimate ± SE	*p*-Value *	Estimate ± SE	*p*-Value *
Footrot score				
0	0		0	
1	0.1338 ± 0.04990	**0.0075**	0.1454 ± 0.0395	**0.0003**
2	0.3546 ± 0.05982	**<0.0001**	0.3561 ± 0.0471	**<0.0001**
3–5	0.4209 ± 0.07450	**<0.0001**	0.4036 ± 0.0578	**<0.0001**
Serogroup				
A	−0.1911 ± 0.0501	**0.0001**	−0.0899 ± 0.0411	**0.0307**
B	−0.0449 ± 0.0521	0.3886	0.0159 ± 0.0420	0.7058
C	−0.1915 ± 0.0595	**0.0013**	−0.1164 ± 0.0494	**0.0188**
D	−0.0131 ± 0.1984	0.9474	−0.0394 ± 0.1514	0.7949
E	−0.0021 ± 0.0987	0.9831	0.0899 ± 0.0793	0.2577
F	0.2188 ± 0.1124	0.0519	0.0499 ± 0.0916	0.5861
G	0.1280 ± 0.0638	**0.0453**	0.0741 ± 0.0519	0.1535
H	−0.0121 ± 0.0613	0.8432	0.0095 ± 0.0488	0.8456
I	−0.1575 ± 0.1220	0.1973	−0.1092 ± 0.0997	0.2738

* significant *p*-Values are in bold.

## Data Availability

All necessary information needed to support the results can be found in the manuscript or are available from the corresponding author upon reasonable request.

## Supplementary Materials

The following are available online at https://www.mdpi.com/article/10.3390/ani12060753/s1: Figure S1. Visualization of serogroup A on a gel. Table S1: Footrot scoring system according to the Swiss Consulting and Health Service for Small Ruminants. Table S2: *p*-values of F-tests using a generalized mixed linear model with flock within region as the random effect for the number of serogroups per animal (N serogroup) and prevalence of each serogroup with each one of the following effects: D. nodosus strains, footrot score, region, herdbook member, breed, flock size, goats on the form, cattle on the farm, horses or donkeys on the farm, treatment of diseased sheep with antibiotics for footrot within the last 12 months, treatment of diseased sheep with footbaths for footrot within the last 12 months, treatment of diseased sheep with vaccines for footrot within the last 12 months, treatment of diseased sheep with antibiotics for footrot within the last 3–10 years, treatment of diseased sheep with footbaths for footrot within the last 3–10 years, treatment of diseased sheep with vaccines for footrot within the last 3–10 years and number of animals tested for serogroups. Table S3: *p*-values of F-tests and the relative proportion of variance between flocks (flock variance in %) for the final generalized mixed linear model for the number of serogroups per animal (N serogroup) and prevalence of each serogroup. Table S4: Distribution of the serogroups by the three different regions of Germany at the flock level.
