# Supplementary material for: Serological Diversity of Dichelobacter nodosus in German Sheep Flocks
Source: Animals (Basel). 2022 Mar 17;12(6):753. doi: 10.3390/ani12060753 (PMC8944645; doi:10.3390/ani12060753)
Supplement: Supplementary file 1 [file animals-12-00753-s001.zip › 11_03_22_Budnik_Supplementary Material rev-bud.pdf]

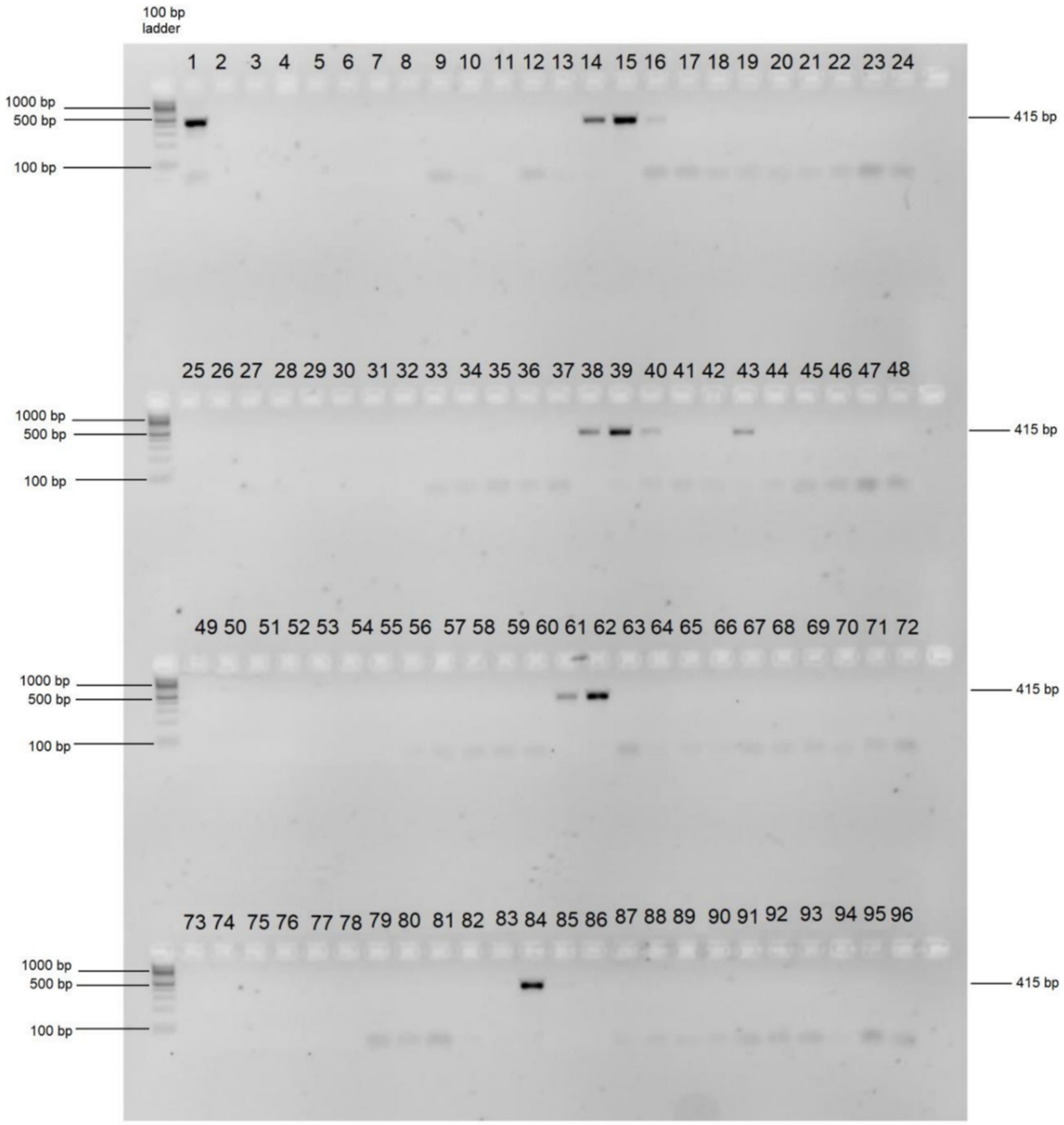

Figure S1. Visualization of serogroup A on gel. Use of a 100 basepair ladder. 1 = positive control; 2-95 = samples; 96 = water. PCR product of serogroup A with a size of 415 basepairs.

Table S1. Footrot scoring system according to the Swiss Consulting and Health Service for Small Ruminants [1].

Footrot score	Clinical signs
0	Healthy interdigital space and claw
1	Limited mild interdigital dermatitis, loss of hair, redness
2	More extensive interdigital dermatitis, foul smell
3	Severe interdigital dermatitis, Separation of hoof horn and dermis on the axial wall
4	Separation of hoof horn and dermis extends to the sole and the abaxial wall
5	Separation of hoof horn and dermis extends to the toe, potential loss of hoof horn

Table S2. P-values of F-tests using a generalized mixed linear model with flock within region as random effect for number of serogroups per animal (N serogroup) and prevalence of each serogroup with each one of the following effects: *D. nodosus* strains, footrot score, region, herdbook member, breed, flock size, goats on the form, cattle on the farm, horses or donkeys on the farm, treatment of diseased sheep with antibiotics for footrot within the last 12 months, treatment of diseased sheep with footbaths for footrot within the last 12 months, treatment of diseased sheep with vaccines for footrot within the last 12 months, treatment of diseased sheep with antibiotics for footrot within the last 3-10 years, treatment of diseased sheep with footbaths for footrot within the last 3-10 years, treatment of diseased sheep with vaccines for footrot within the last 3-10 years and number of animals tested for serogroups.

**Source of variation**	**Serogroup**									**N serogroup**
	**A**	**B**	**C**	**D**	**E**	**F**	**G**	**H**	**I**	
***D. nodosus* strains**	0.5766	0.2440	0.8411	0.7943	0.8036	0.9480	0.0719	**0.0385**	**0.0223**	0.2363
**Load of *D. nodosus***	**0.0032**	0.4447	**0.0013**	0.3262	0.9301	**0.0252**	**0.0455**	0.5328	0.2396	**0.0009**
**Footrot score**	**<0.0001**	**0.0438**	0.0859	0.1858	**0.0099**	0.9861	0.0947	**<0.0001**	0.2613	0.9164
**Region**	**0.0029**	0.5901	0.3056	0.8628	0.3603	0.3488	0.2480	0.6140	0.9670	**0.0098**
**Herdbook**	0.5087	0.9160	0.1523	0.5539	0.1660	0.8176	0.0824	0.6887	0.4125	0.3569
**Sheep breed**	**0.0029**	**0.0003**	**0.0091**	0.9947	0.7385	0.7566	0.8881	0.8845	0.9941	0.4465
**Flock size**	0.9474	0.2153	0.5163	0.5333	**0.0054**	0.1717	0.4004	0.4791	0.1533	0.1911
**Goats on farm**	0.7594	0.5404	0.0647	0.1438	0.0963	0.6707	0.8002	0.7954	0.8419	0.7192
**Cattle on farm**	0.1816	0.1204	0.6881	0.7676	0.2012	0.2190	0.6224	0.8254	0.8754	0.0761
**Horses on farm**	0.3767	0.1826	0.0523	0.8098	0.1619	0.5974	0.0690	0.9522	0.9978	0.9596
**Donkeys on farm**	0.5050	0.9891	0.3580	0.3679	0.9004	0.9896	0.0295	0.1790	0.4711	0.1889
**Footrot <12 months**	0.1517	0.4647	0.0998	0.8252	0.6330	0.3304	0.8270	0.8582	0.4174	0.6231
**Antibiotics <12 months**	**0.0067**	0.7343	0.4280	0.5445	0.3673	0.4468	0.8731	**0.0037**	0.3257	**0.0001**
**Footbaths <12 months**	0.5093	0.5705	0.5956	0.6568	0.7296	0.8976	0.9165	0.1402	0.8780	0.0867
**Vaccines <12 months**	0.6966	0.1921	0.1642	0.3120	0.2452	0.1179	0.6109	0.1105	0.6616	0.1828
**Footrot <3-10 years**	0.1038	0.4278	0.1888	0.6515	0.9249	0.9266	0.7832	0.5626	0.8775	0.5833
**Antibiotics 3-10 years**	0.7787	0.0718	0.9134	0.8495	0.4981	**0.0260**	0.7239	0.2102	0.8206	0.3748
**Footbaths 3-10 years**	0.2142	0.8006	0.5529	0.8075	0.1193	**0.0331**	0.9878	0.2207	0.7985	0.2612
**Vaccines 3-10 years**	0.2178	0.8832	0.8151	0.8212	0.3006	0.2427	0.6268	0.4505	0.8643	0.6257
**Animals tested per flock**	0.1222	0.1030	0.9744	0.6531	0.7520	0.8448	0.2066	0.6685	0.8043	0.4827
**Flock within region**	**<0.0001**	**<0.0001**	**<0.0001**	**<0.0001**	**<0.0001**	**<0.0001**	**<0.0001**	**<0.0001**	**<0.0001**	**<0.0001**

Table S3. P-values of F tests and relative proportion of variance between flocks (flock variance in %) for the final generalized mixed linear model for number of serogroups per animal (N serogroup) and prevalence of each serogroup.

Source of variation	Serogroup									N serogroup
	A	B	C	D	E	F	G	H	I	
D. nodosus strains								**0.0443**	**0.0223**	
Load of *D. nodosus*	**0.0009**		**0.0006**			**0.0367**	**0.0455**			**0.0017**
Footrot score	**<0.0001**	**0.0463**			**0.0257**			**<0.0001**		
Region	**0.0029**									**0.0258**
Herdbook										
Sheep breed	**0.0050**	**0.0003**	**0.0137**							
Flock size					**0.0162**					
Goats on farm										
Cattle on farm										
Horses on farm										
Donkeys on farm										
Footrot <12 months										
Antibiotics <12 months	**0.0177**							0.0594		**0.0003**
Footbaths <12 months										
Vaccines <12 months										
Footrot <3-10 years										
Antibiotics 3-10 years						0.0748				
Footbaths 3-10 years						**0.0266**				
Vaccines 3-10 years										
Animals tested per flock										
Flock within region	**<0.0001**	**<0.0001**	**<0.0001**	**<0.0001**	**<0.0001**	**<0.0001**	**<0.0001**	**<0.0001**	**<0.0001**	**<0.0001**
Flock variance (%)	**48.9**	**54.6**	**58.5**	**32.9**	**31.3**	**59.4**	**50.8**	**66.1**	**50.5**	**21.9**

Table S4. Flock-level results for the 83 flocks by region, number of animals per flock, number of animals serotyped, frequency of untypeable samples, number of samples by serogroup A-I and untypeable serogroup, frequency of samples positive for *aprB2* only, frequency of samples positive for both *aprV2* and *aprB2*, frequency of samples positive for *aprV2* only, average footrot score per flock, number of animals with the respective footrot score.

Table S5. Distribution of the serogroups by the three different regions of Germany on flock level. Number and percentage of flocks with the respective serogroup. The percentages refer to the number of flocks in the respective region.

Region	Number of flocks	No. of serotyped animals	No. (%) of flocks where the respective serogroup was detected								
			A	B	C	D	E	F	G	H	I
North	52	487	36 (69.23)	33 (63.46)	24 (46.15)	2 (3.85)	9 (17.31)	9 (17.31)	17 (32.69)	24 (46.15)	5 (9.62)
East	14	157	10 (71.43)	7 (50.00)	8 (57.14)	2 (14.29)	2 (14.29)	3 (21.43)	6 (42.86)	6 (42.86)	1 (7.14)
South	17	175	7 (41.18)	13 (76.47)	8 (47.06)	1 (5.88)	4 (23.53)	2 (11.77)	8 (47.06)	8 (47.06)	1 (5.88)

Table S6. Serogroup combinations on flock level with declaration of times determined.

No. of serogroups per flock	No. of flocks	Serogroup (combination)	Times determined
1	10	A	2
		B	2
		C	2
		F	1
		G	1
		H	2
2	16	A, B	2
		A, C	2
		B, C	2
		B, G	1
		B, H	4
		C, G	1
		A, H	3
		C, F	1
3	28	A, B, C	3
		B, F, G	1
		A, B, H	7
		A, B, G	4
		A, C, H	1
		A, D, G	1
		B, G, H	1
		A, C, G	4
		A, D, H	1

		B, C, H	2
		B, C, G	1
		A, E, H	1
		E, G, I	1
4	19	A, E, F, G	1
		A, B, C, H	3
		B, E, H, I	1
		A, B, E, H	2
		B, C, E, G	1
		A, B, C, G	3
		B, C, F, G	1
		A, C, E, F	2
		A, B, G, I	1
		B, C, G, H	1
		A, B, C, F	1
		B, C, F, H	1
		D, F, H, I	1
5	6	B, C, F, G, H	1
		A, B, D, H, I	1
		A, B, E, F, H	1
		A, B, C, E, F	1
		A, B, C, G, H	1
		A, C, G, H, I	1
6	4	A, B, C, E, G, H	1
		A, B, C, E, F, G	1
		A, C, D, E, G, H	1
		A, B, C, E, G, I	1

References

1. Aepli, M.; Bähler, S.; Dürr, S.; Grieder, S.; Härdi, C.; Kuhlgatz, C.; Lüchinger, R.; Mengelt, R.; Rediger, M.; Schüpbach, G.; et al. Ökonomische Auswirkungen der Moderhinke und Kosten-Nutzen-Analyse einer Bekämpfung der Moderhinke in der Schweizer Schafpopulation. *Ed.; Bundesamt für Lebensmittelsicherheit und Veterinärwesen (BLV) und Bundesamt für Landwirtschaft (BLW), ETH Zurich: Zurich, Switzerland* **2016**.
